# Combination of low doses of mirtazapine plus venlafaxine produces antidepressant-like effects in rats, without affecting male or female sexual behavior

**DOI:** 10.1007/s00213-024-06661-2

**Published:** 2024-08-07

**Authors:** Adriana Álvarez-Silva, Gabriela Rodríguez-Manzo, Rebeca Reyes, Alonso Fernández-Guasti

**Affiliations:** Departamento de Farmacobiología, Sede sur, Cinvestav, Mexico

**Keywords:** Mirtazapine/Venlafaxine combined treatment, Antidepressant-like effect, Forced swim test, Chronic mild stress, Male and female sexual behavior

## Abstract

**Rationale:**

Pharmacological treatments for depression are not always effective and produce unwanted side effects. Male and female sexual dysfunction is one of these side effects, which can lead to treatment withdrawal. Combination of two antidepressants with different mechanisms of action, like mirtazapine (MTZ) and venlafaxine (VLF) have been shown to be effective for treatment-resistant depression in humans. Combination of low doses of these drugs may still exert antidepressant-like effects without altering sexual behavior.

**Objectives:**

To investigate the potential antidepressant-like effect of the chronic administration of low doses of MTZ plus VLF combined, as well as its impact on male and female sexual behavior in rats.

**Methods:**

The antidepressant-like effect of a 14-day treatment with combinations of MTZ plus VLF (0/0, 2.5/3.75 or 5/7.5 mg/kg) was assessed in young adult male and female rats in the forced swim test (FST). The 5/7.5 mg/kg MTZ/VLF combination was also tested in the chronic mild stress (CMS) test, in both males and females treated for 21 days. The sexual effects of this last treatment were assessed in sexually experienced males and in gonadally-intact females during proestrus.

**Results:**

The 5/7.5 mg/kg MTZ/VLF combination produced an antidepressant-like effect in the FST and reversed the CMS-induced anhedonia in both male and female rats. This combination did not alter male sexual behavior, female proceptive and receptive behaviors or the regularity of the estrous cycle.

**Conclusion:**

The combination of low doses of MTZ and VLF might be a promising therapeutic alternative to treat depression without affecting the sexual response.

## Introduction

Depression is a common and severe psychiatric disorder affecting approximately 280 million people around the world (WHO 2023). One of the worst consequences of depression is suicide, which causes 700,000 deaths every year. There are many pharmacological treatment options for depression, however, more than half of the patients do not achieve depressive symptoms’ remission after treatment with a single antidepressant (Golden et al. 2002). Moreover, in patients showing an adequate response, residual symptoms like anxiety or insomnia may persist (Nierenberg et al. 1999; McClintock et al. 2011); these residual symptoms may lead to depression relapse.

Combining antidepressants of different pharmacological classes has been suggested as a strategy to improve the response to treatment. An example of this is the combination of mirtazapine (MTZ), a noradrenergic and specific serotonergic agent (NaSSA), and the serotonin and noradrenaline reuptake inhibitor (SNRI), venlafaxine (VLF). Some studies have reported a high efficacy of the combination of therapeutic doses of these antidepressants from the beginning of treatment (McGrath et al. 2006), or as by the addition of MTZ when VLF monotherapy shows a poor response (Carpenter et al. 2002; Aydemir et al. 2009; Kessler et al. 2018; Navarro et al. 2019). However, no clinical study has explored whether the combination of lower doses of these antidepressants might be therapeutically useful. Besides, gender-related differences in the antidepressant response have been described (Frackiewicz et al. 2000; Kornstein et al. 2000; Berlanga and Flores-Ramos 2006); for example, women exhibit a higher antidepressant response to selective serotonin reuptake inhibitors (SSRIs) (Keers and Aitchison 2010; Sramek and Cutler 2011) than to noradrenaline reuptake inhibitors (NRIs), whereas men respond well to both SSRIs and NRIs (Berlanga and Flores Ramos 2006), but also to tricyclic antidepressants (Kornstein et al. 2000; Sramek and Cutler 2011). Therefore, comparison of the effects of MTZ plus VLF combined between sexes would be relevant.

Several animal models are currently used for the study of depression. However, only few meet the main validity criteria: face, predictive and construct validity (Duman 2010). One of these is the chronic mild stress (CMS) model, in which animals are exposed to different mild stressors over a period of some weeks and the animal’s responsiveness to natural rewarding stimuli, such as sex or sucrose preference, is analyzed to determine the presence of one of the core symptoms of depression, anhedonia (Willner 1997). Also, the CMS produces a wide variety of somatic and behavioral disturbances that are comparable to those presented by depressed patients (Cheeta et al. 1997; Papp 2012; Wiborg 2013), many of which are reversed after the chronic, but not acute, treatment with antidepressants (Papp et al. 1996). The main disadvantage of the CMS model is the long time and effort required to obtain results (Markov and Novosadova 2022). Zhang et al. (2010) and Barbar et al. (2022) reported an increase in sucrose preference in male rats with CMS-induced anhedonia after the administration of MTZ. Regarding VLF, Zhang et al. (2010) and Xing et al. (2013) also found an antidepressant-like effect in stressed male and female rats. To our knowledge no study has explored the effect of combining these two antidepressants in the CMS model.

On the other hand, the forced swim test (FST) is a widely used, rapid and easy to perform method to assess the efficacy of antidepressant treatments. This test is based on the behaviors displayed by the animal in an unescapable situation: forced swimming. Initially, the animal displays active behaviors such as swimming, diving or climbing, but eventually it adopts an immobility posture, which is considered to reflect behavioral despair (Yankelevitch-Yahav et al. 2015). Although immobility in the FST has recently been considered an adaptive response, rather than a valid measure of despair (Molendijk and de Kloet 2015), this test is useful as a screening method to identify the antidepressant properties of new drugs, showing a high sensitivity to a broad range of antidepressants (Slattery and Cryan 2012). Using this test, the antidepressant-like effect of MTZ has been confirmed in several studies (Rénéric et al. 2002a, 2002b; Alvarez Silva and Fernández Guasti 2019; Barbar et al. 2022). In a previous report, we found that 40 mg/kg MTZ decreased immobility in the FST in both male and female rats (Alvarez Silva and Fernández-Guasti 2020); however, this dose caused significant sedation, probably due to H1-histaminergic receptor antagonism (Nutt 2002). VLF, on the other hand, was effective in reducing immobility in the FST at a dose of 60 mg/kg in male and female rats (Alvarez Silva and Fernández-Guasti 2020), while in other studies, antidepressant-like effects of VLF have been observed with a wide range of doses (10–80 mg/kg) (Rénéric and Lucki 1998; Rogóz et al. 2002). There is a single study reporting the effects of the MTZ-VLF combination in the FST. In that study, we showed that low doses of MTZ and VLF reduced immobility and increased swimming in ovariectomized, hormonally primed female rats (Alvarez Silva and Fernández-Guasti 2019).

One of the main side-effects of antidepressants are related to sexual behavior. In general, antidepressants produce a wide range of sexual side-effects that are related to their target neurotransmitter systems. Thus, antidepressants acting at the serotonergic system decrease rodent masculine (Olivier and Olivier 2019) and feminine (Clayton 2003; Clayton et al. 2014) sexual behavior, whereas antidepressants acting at catecholaminergic systems have the opposite effect (Hull et al. 2004; Olivier and Olivier 2019). Previous studies in male rats have shown that MTZ enhanced sexual motivation (Benelli et al. 2004), while VLF negatively affected sexual behavior (Bijlsma et al. 2014). Regarding female sexual behavior there is a single report showing that, when given alone, these drugs, at doses with antidepressant-like effect, reduced female sexual behavior (Alvarez Silva and Fernández-Guasti 2019). Conversely, the sub-chronic treatment with a combination of low doses of MTZ and VLF (also with antidepressant-like effect) did not alter female sexual behavior (Alvarez Silva and Fernández-Guasti 2019).

In humans, the estimated incidence of antidepressant-related sexual dysfunction is close to 40%, although the exact number is unknown (Rothschild 2000). In men, the most frequent sexual side-effects of antidepressants are erection and ejaculation impairments (Kennedy and Rizvi 2009), whereas in women sexual desire, arousal and orgasm are the most affected (Lorenz et al. 2016). These side-effects impact the patients’ quality of life and can lead to treatment withdrawal. The antidepressant drugs that consistently produce sexual dysfunction are those with serotonergic effects, like the SSRIs, fluoxetine or sertraline, and the SNRIs showing higher affinity for the 5-HT transporter, like duloxetine or VLF (Adachi et al. 2010; Higgins et al. 2010; Rothmore 2020). Finally, a low sexual dysfunction incidence has been observed with antidepressants blocking 5-HT2 receptors, like MTZ and nefazodone, and the noradrenaline and dopamine reuptake inhibitor (NDRI), bupropion (Rothschild 2000).

In summary, sub-chronic treatment with the combination of MTZ plus VLF produces an antidepressant-like effect in the FST in male and female rats, while lacking sexual effects in ovariectomized, steroid-primed female rats. However, it is unknown if chronic treatment, as used in clinical practice, of the combination of low doses of MTZ plus VLF produces an antidepressant-like effect in a well-sustained animal model of depression like the CMS, and if such a combination alters the display of sexual behavior in intact (non-ovariectomized) females and in male rats.

## Materials and methods

### Animals

Young adult (3–4 months old) female (180–250 g) and male (300–350 g) Wistar rats produced in our animal facilities were used in this study. All animals were kept under 12 h/12 h inverted light–dark cycle conditions, with lights on at 22:00 h, in a room with controlled temperature (21 ± 1 °C) and humidity. Five to six same-sex animals per cage were housed with ad libitum access to water and chow. All experimental procedures were performed in accordance with the Mexican Official Norm for animal care and handling (NOM-062-ZOO-1999) and approved by the Institutional Committee for the Care and Use of Laboratory Animals (CICUAL; protocol number 0025–13).

### Drugs

Mirtazapine (MTZ) (Bioquimed Mexico City) was dissolved in physiological saline with 0.5% acetic acid. Venlafaxine (VLF) hydrochloride (Bioquimed Mexico City) was dissolved in physiological saline. MTZ was used at concentrations of 1.25 and 2.5 mg/ml and VLF at 1.875 and 3.75 mg/ml. Both drugs were intraperitoneally administered in a volume of 2 ml/kg.

*Experiment 1. Assessment of antidepressant-like effects of chronic treatment with two combinations of doses of mirtazapine and venlafaxine in the FST in male and female rats*

The FST consisted of placing individual male or female rats into glass cylinders (45 cm tall × 20 cm diameter) filled with water 30 cm deep (Detke et al. 1995). Two swimming sessions were conducted: a 15-min pre-test, followed 14 days later by a 5-min test (Álvarez Silva and Fernández-Guasti, 2019; Estrada-Camarena et al. 2008). Drug treatments were administered between these sessions. After both sessions, rats were removed from the cylinders, dried with towels, and placed on warm cages, and later returned to their home cages. Test sessions were videotaped for later scoring of three different behaviors: immobility, swimming and climbing. The behaviors were scored every 5-s, so that 60 counts were obtained along the 5-min test.

Independent groups of male and female rats were administered with a combination of 2.5/3.75 or 5/7.5 mg/kg of MTZ and VLF, respectively (see timeline for Experiment 1). Male and female rats were divided into three groups (8 animals each): one group for each antidepressant combination, and a group receiving the vehicles. Daily administrations along 14 days were made during the first 4 h of the dark phase of the cycle. Several previous clinical reports indicate that two to four weeks of treatment with such combination of antidepressants is the minimum lapse required to observe antidepressant effects (Furukawa et al. 2019; Mahli, et al. 2008; Mc Grath et al. 2006; Carpenter et al. 2002). In this first experiment we explored if two weeks of treatment produced an antidepressant-like effect. The doses of the antidepressant combinations were chosen based on previous experiments showing their effectiveness in reducing depressive-like behaviors following a sub-chronic treatment (Alvarez Silva and Fernández-Guasti 2019).

### Open-field test

Immediately after the FST (see timeline for Experiment 1), all rats were individually tested for locomotor activity with an electronic actimeter (Panlab LE 8825) that consisted of a polypropylene cage (45 × 45 × 20 cm) surrounded by two infrared beam frames. The rat was placed into the center of the cage, left for five minutes, and the general activity, stereotyped movements and rearings were recorded. At the end of each individual test, the box was cleaned with a 70% alcohol solution to eliminate odor traces.
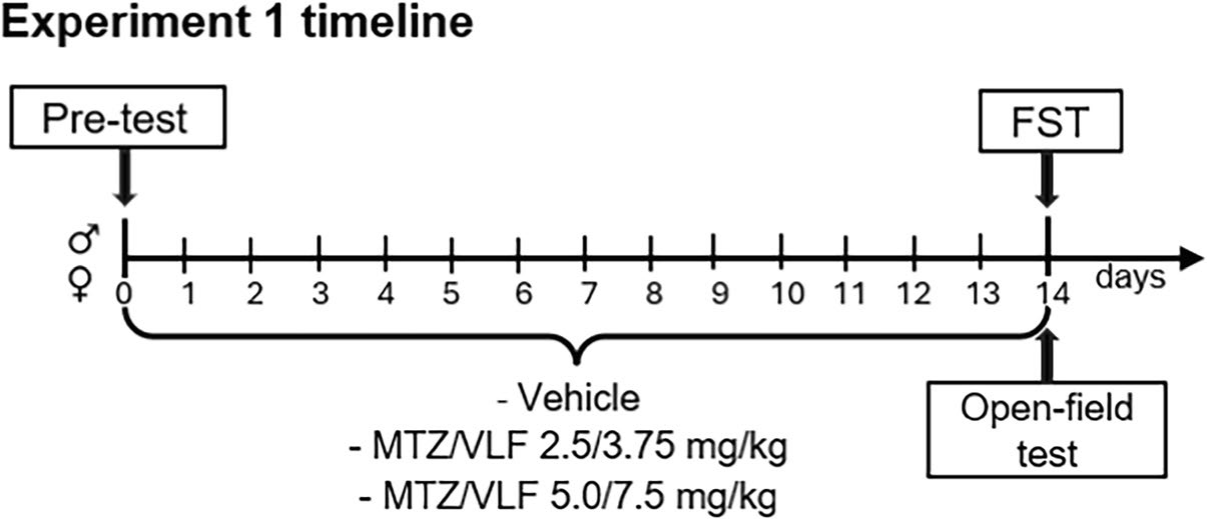


*Experiment 2. Evaluation of the antidepressant-like effect of chronic treatment with a combination of mirtazapine and venlafaxine on chronic-mild stress-induced anhedonia in male and female rats.*

The CMS analyzes the reduction in the rewarding properties of sucrose consumption after the exposure to various mild stressors for a prolonged period as an indicator of anhedonia, a central symptom of depression.

Male and female rats were housed individually. The groups subjected to the CMS protocol were placed in an isolated room, while control non-stressed rats were housed under standard vivarium conditions.

### Sucrose consumption

To control for neophobia all rats (male and female) were allowed to adapt to the taste of a sucrose solution (1%) for two consecutive weeks (see timeline for Experiment 2). During this period, the regular water bottle was substituted by a bottle containing sucrose solution for one hour, every day, at the beginning of the dark phase of the cycle (10 AM). At the end of this time, the baseline sucrose consumption was determined. Before the measurement, rats were water- and food-deprived for 20 h and thereafter presented with two bottles for 1 h: one containing sucrose solution and the other tap water. Fluid (sucrose solution or tap water) intake was measured by weighing the bottles before and after the test. To determine that rats developed sucrose preference, the intake of sucrose solution had to be at least twice the volume of water consumed and ≥ 4 g. Only the animals that fulfilled these criteria were included in the study.

### CMS paradigm

In female rats, the CMS paradigm that effectively reduced the sucrose intake included the exposure to the following stressors for 3 weeks: white noise (~ 90 dB), continuous light, overcrowding (2–3 animals per individual cage), stroboscopic light (300 flashes/min), 45° cage tilt along the vertical axis, soiled cage (250 ml water spilled into bedding), and water deprivation (Table 1). This schedule has been reported to induce anhedonia in young adult female (Récamier-Carballo et al. 2012) and old males, but not in young male rats (Herrera-Pérez et al. 2008). In the present study we conducted in a pilot experiment the schedule of stressors that effectively reduced sucrose intake in young females and found no effect in males. On these bases we decided to expose the young male rats to a modified CMS timetable. In this case, the white noise and continuous light stressors were substituted by ultrasonic noise of an intensity varying between 20–45 kHz (see Table 1). Studies have shown that the continuous exposure to these ultrasonic frequencies induces depressive-like behaviors in rats and mice (Morozova et al. 2016; Strekalova et al. 2018; Pavlov et al. 2019). Moreover, the male rats were exposed to the stress protocol for a longer period than females, 4 weeks (see timeline for Experiment 2, males). Control groups of non-stressed animals were maintained for the same period under comparable handling and housing conditions than the stressed animals.

**Table 1:** Chronic mild stress (CMS) schedule used to induce anhedonia in female and male rats

Time	1st Wed	Thu	Fri	Sat	Sun	Mon	Tue	Wed
7–8			SC/(CL) US	WD			OC/(CL) US	FD/WD
8–9			SC/(CL) US	WD			CT/(CL) US	FD/WD
9–10			CL	WD			CT/(CL) US	FD/WD
10–11	BASELINE		SL	(WN) US			CT/(CL) US	**TEST**
11–12		OC	SL	(WN) US			CT/(CL) US	
12–13		OC	SL	(WN) US		SL	CT	
13–14	(WN) US	OC	SL	(WN) US		SL	CT	(WN) US
14–15	(WN) US	OC	SL			SL	FD/WD	(WN) US
15–16	(WN) US	OC/(CL) US				SL	FD/WD	(WN) US
16–17		SC/(CL) US	WD			SL	FD/WD	
17–18		SC/(CL) US	WD			OC/(CL) US	FD/WD	
18–19		SC/(CL) US	WD			OC/(CL) US	FD/WD	
19–20		SC/(CL) US	WD			OC/(CL) US	FD/WD	
20–7		SC/(CL) US	WD			OC/(CL) US	FD/WD	

The stressors employed during the CMS protocol were WN: white noise (~ 90 dB), OC: overcrowding (2–3 rats per cage), CL: continuous light, SC: soiled cage (250 ml water spilled into bedding), SL: stroboscopic light (300 flashes/min), WD: water deprivation, CT: cage tilt (45°), FD: food deprivation. Abbreviations in parenthesis show the stressors that were substituted by the ultrasonic sound (US) in male rats

In all groups, the rats' sucrose solution and tap water consumption was determined weekly after a 20 h period of water and food deprivation (TEST, Table 1, timeline for Experiment 2). This evaluation consisted of 1-h exposure to two bottles: one with sucrose solution and the other containing tap water. The anhedonic state generated by the CMS was reflected in a reduced sucrose consumption of at least 2 g in two successive evaluations, or at least 5 g between the baseline and the final tests (Herrera-Pérez et al. 2008).

Once sucrose consumption decreased in the stressed animals to meet the anhedonia criteria (three weeks for females and four weeks for males, see timeline below), the animals were subdivided into two groups, each one receiving the combination of MTZ and VLF 5/7.5 mg/kg or their vehicles. Non-stressed animals were also subdivided into these two treatment groups after three weeks of standard housing. During the treatment period, rats of the CMS groups continued to be exposed to the stressors, while control unstressed rats remained in the vivarium. All groups were tested weekly to determine the effect of the antidepressant combination, or their vehicles, on the sucrose consumption. In the stressed groups, treatments were interrupted in both male and female rats, once the sucrose intake returned to pre-stress basal levels.


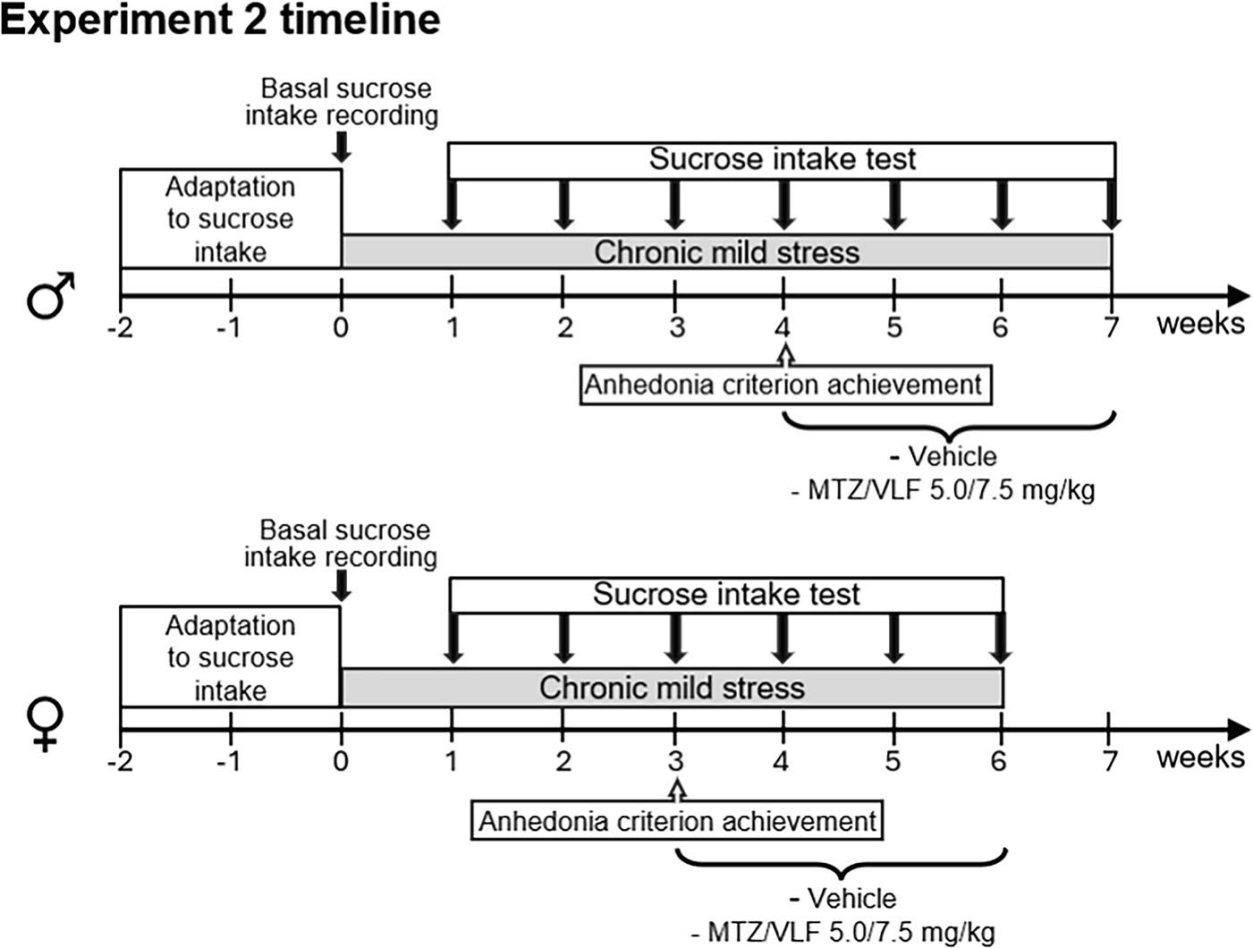


*Experiment 3. Analysis of the sexual behavior after chronic treatment with the combination of mirtazapine and venlafaxine in male and female rats*

### Male sexual behavior

Male rats underwent 3 to 5 sexual training sessions with receptive females, conducted every other day, to render them sexually experienced (Lucio et al. 2022; Rodríguez-Manzo and Canseco-Alba 2023). Males were considered sexually experienced when attaining an ejaculation latency of 15 min or less in three training sessions. For the sexual behavior tests, male rats were introduced into a mating arena (50 cm diameter × 40 cm height) and a 5-min habituation period was allowed before introducing a sexually receptive female. Sexual interaction was permitted during 30 min and sessions were videotaped for later analysis. The copulatory parameters scored were the latencies to the first mount or intromission, the number of pre-ejaculatory mounts and intromissions, the ejaculation latency (time between the first intromission and ejaculation), the inter-intromission interval (ejaculation latency divided by the number of intromissions), the post-ejaculatory interval (time from ejaculation until the appearance of the first intromission of the next series) and the number of ejaculations displayed (Rodríguez-Manzo and Canseco-Alba 2023).

Independent groups (8 animals each) of male rats were treated with a combination of the antidepressants MTZ (5 mg/kg) plus VLF (7.5 mg/kg) or their vehicles. Daily administrations were made during the first 4 h of the dark phase of the cycle (see Experiment 3 timeline, males). Based on the results of the Experiment 2, males were tested for sexual behavior after treatment with MTZ plus VLF for 3 weeks (21 days).

### Female sexual behavior

To monitor estrous cyclicity female rats were subjected to daily sampling of vaginal smears for 14 days before the beginning of the experiment, and until the last day of drug administration (see Experiment 3 timeline, females). The estrous cycle phases were determined according to the description of Marcondes et al. (2002).

For the sexual behavior tests, female rats were selected during the late proestrus phase of the estrous cycle. A sexually experienced male rat was introduced into the cylindrical mating arena five minutes before introducing the female. Thereafter, the animals were allowed to sexually interact until the female received 10 mounts from the male. Sexual activity was videotaped for further analysis of proceptive and receptive behaviors. The proceptive behaviors recorded were hopping, darting and ear wiggling (for descriptions see Erskine 1989). To measure receptivity, the intensity of lordosis (IL) was assessed using a four-point scale from 0 to 3 as described by Hardy and DeBold (1971) and the lordosis quotient (LQ) was determined using the following formula: # of lordosis/10 mounts × 100.

Independent groups (8 animals each) of female rats were treated with a combination of the antidepressants MTZ (5 mg/kg) plus VLF (7.5 mg/kg) or their vehicles. Females were tested after treatment for 21–24 days; the reason for this variation was that females were tested for female sexual behavior under natural conditions, i.e., in late proestrus (see Ventura-Aquino and Fernandez-Guasti 2013 for methodological details and timeline for females below).
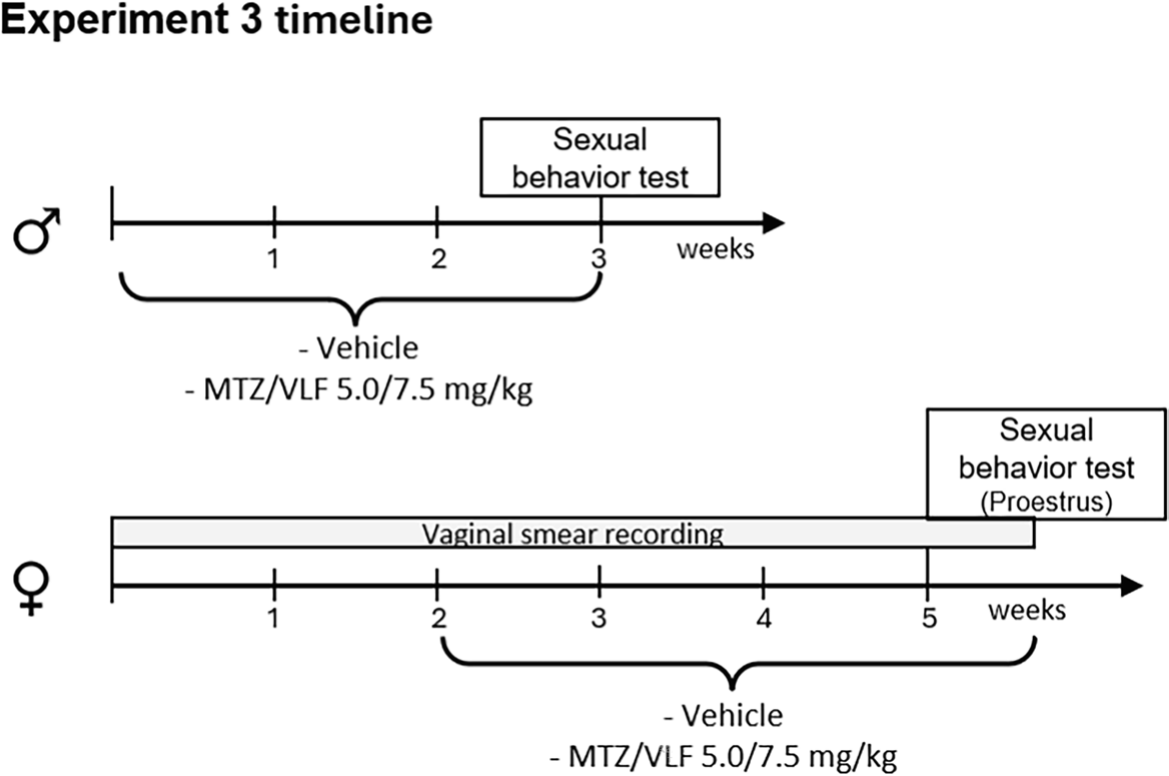


### Statistical analyses

The data of the experimental models of depression and spontaneous motor activity are presented as mean ± standard error of the mean. FST data were analyzed using the Kruskal–Wallis ANOVA followed by Dunn’s test. Non-parametric statistics was used because not all data passed the normality tests. Sex comparisons were made using a two-way ANOVA followed by Tukey test. CMS data were analyzed using a repeated measures two-way ANOVA followed by Tukey test. Paired comparisons were conducted with t tests. Sexual behavior results also did not pass normality and equal variance tests; therefore, they were analyzed with non-parametric statistics and are presented as medians ± interquartile ranges. Specifically, the comparisons between control and drug-treated groups were made by means of the Mann–Whitney U test. Differences were considered statistically significant with a *p* value ≤ 0.05.

## Results

*Experiment 1. Assessment of antidepressant-like effects of chronic treatment with two combinations of doses of mirtazapine and venlafaxine in the FST in male and female rats*

**Fig. 1:**
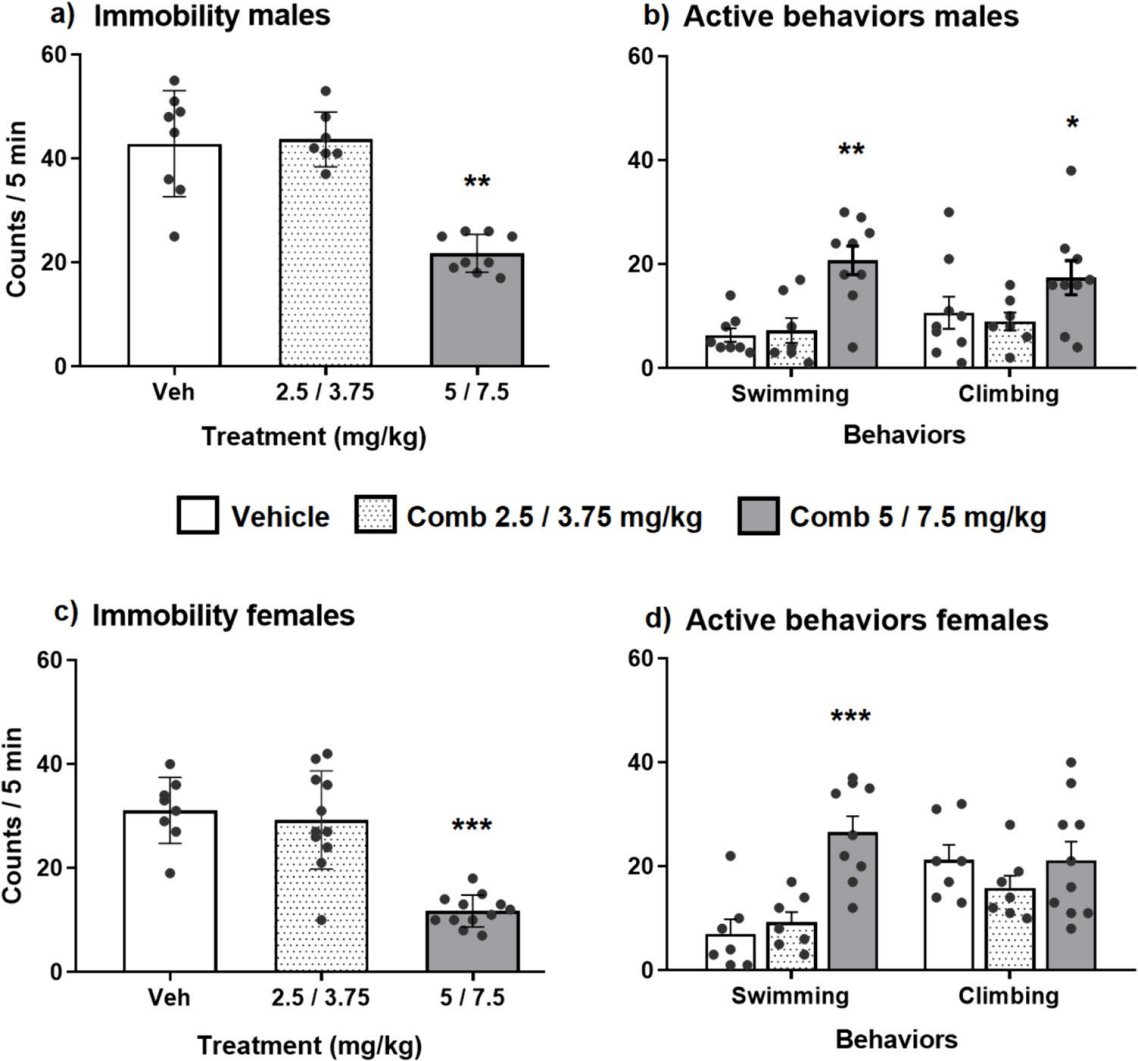
Effect chronic treatment with MTZ/VLF combinations on the forced swim test. Immobility (**a**, **c**) and active behaviors (swimming and climbing) (**b**, **d**) of male and female rats subjected to the FST after chronic treatment (14 days) with two different combinations of MTZ plus VLF (2.5/3.75 or 5/7.5 mg/kg, respectively) or their vehicles. Data are expressed as mean ± SEM of the number of counts/5 min. Kruskal–Wallis ANOVA followed by Dunn’s test *P < 0.05; **P < 0.01, *** P < *0.001* vs. vehicle, *n* = 8 per group

The concomitant administration of MTZ (5 mg/kg) plus VLF (7.5 mg/kg) to male rats for 14 days reduced immobility behavior in the FST (Kruskal–Wallis ANOVA H_2_ = 14.84, *p* = 0.006, Dunn’s, *p* = 0.0016), and increased both swimming (Dunn’s, *p* = 0.009) and climbing behaviors (Dunn’s, *p* = 0.05) (Fig. 1a, b). The combination of lower doses of MTZ and VLF (2.5/3.75 mg/kg) did not reduce immobility (Dunn’s, NS) nor modified active behaviors in males (Fig. 1a,b).

In female rats, the combination of MTZ and VLF (5/7.5 mg/kg) for 14 days decreased immobility (Kruskal- Wallis ANOVA H_2_ = 18.56, *p* = 0.0001, Dunn’s *p* = 0.0004) (Fig. 1c) and increased swimming (Dunn’s *p* = 0.0006) but not climbing (Dunn’s, NS) (Fig. 1d). The combination of 2.5/3.75 mg/kg MTZ plus VLF also lacked effects on immobility behavior in females (Dunn’s, NS) (Fig. 1c, d).

Comparison of the antidepressant-like effect of the combination of MTZ and VLF (5/7.5 mg/kg) between males and females was performed with a two-way ANOVA, taking sex and treatments as factors. Results showed a significant effect on immobility for sex and treatment, but not for their interaction (Sex: F_1,39_ = 41.04, *p* < 0.0001; Treatments F_2,39_ = 48.38, *p* < 0.0001; Interaction: F_2,39_ = 0.5686, NS). A sex difference in the basal levels of immobility between males (43 counts/5 min) and females (31 counts/5 min) was also observed. The lack of interaction between sex and treatment indicates a similar antidepressant-like effect of this combination in males and females. Comparison of the active behaviors between sexes showed a difference in climbing behavior in the factors sex and treatment, but not in their interaction (Sex: F_1,39_ = 9.2, *p* = 004; Treatments F_2,39_ = 3.69, *p* = 0.03; Interaction: F_2,39_ = 1.7, NS); while only a difference in the treatment factor was found in swimming behavior (Sex: F_1,39_ = 2.55, NS; Treatments F_2,39_ = 26.31, *p* < 0.0001; Interaction: F_2,39_ = 1.02, NS). Such differences were related to the increase in swimming and climbing observed only in males after this combination (vide supra).

Table 2 shows the spontaneous motor activity displayed by male rats treated with the two different combinations of MTZ and VLF. Only the combination of the higher doses (5/7.5 mg/kg) reduced the spontaneous activity of rats as compared to the control group (Kruskal–Wallis ANOVA, H_2_ = 14.15, *p* = 0.008; Dunn’s *p* = 0.003).

**Table 2:** Locomotor activity of male rats after MTZ plus VLF (2.5/3.75 or 5/7.5 mg/kg) or their vehicles

**Treatments**	Locomotor activity (counts/5 min) (Mean ± SEM)
Vehicles	1931 ± 111
MTZ 2.5/VLF 3.75 mg/kg	1523 ± 113
MTZ 5/ VLF 7.5 mg/kg	1140 ± 51 **

*Kruskal–Wallis ANOVA, *post hoc Dunn’s, ** p < 0.01, n = *8 per group*

*Experiment 2. Evaluation of the antidepressant-like effect of chronic treatment with a combination of mirtazapine and venlafaxine on chronic-mild stress-induced anhedonia in male and female rats.*

### Male rats

**Fig. 2:**
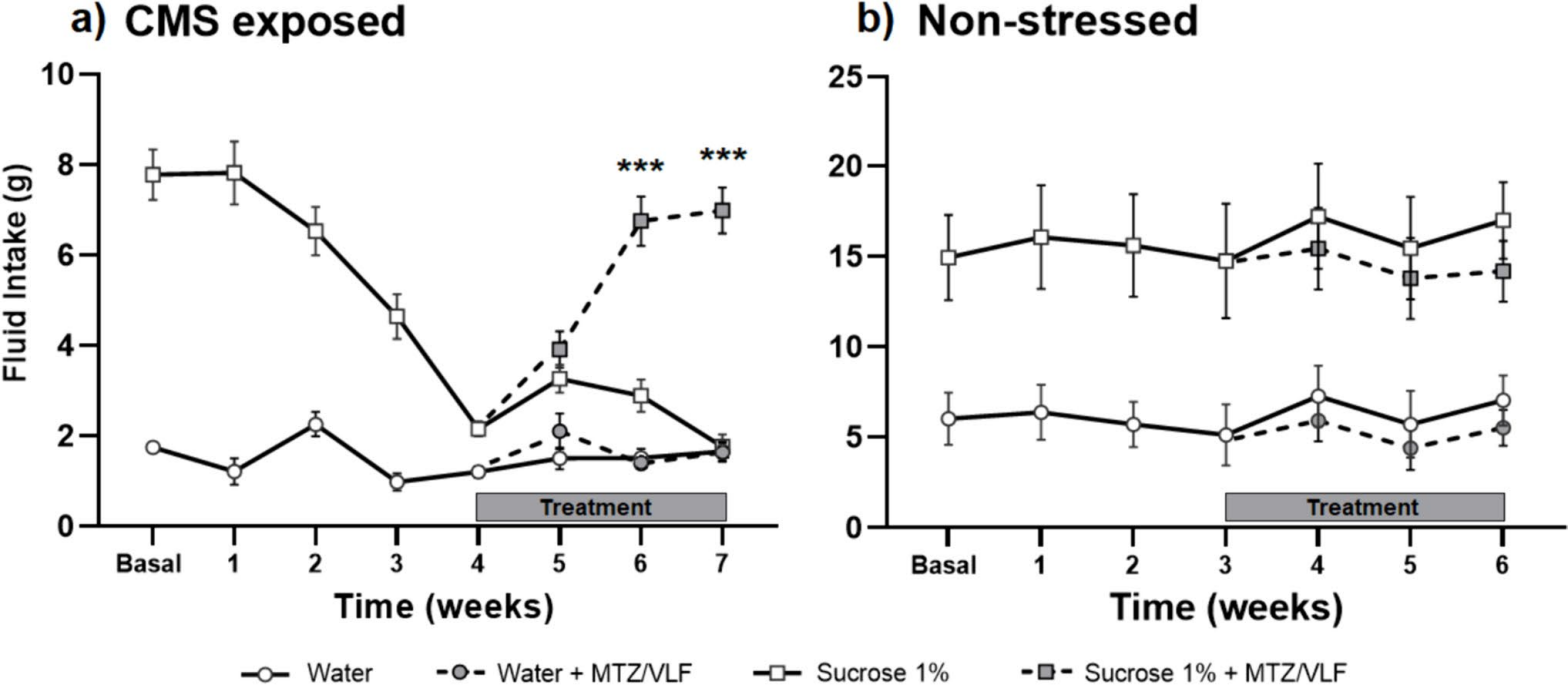
Sucrose intake of male rats that developed anhedonia after CMS in response to MTZ/VLF combined treatment. **a**) Male rats’ water and sucrose solution intake during CMS exposure before and after a 3-week treatment with the combination of MTZ plus VLF (5/7.5 mg/kg) or their vehicles. **b**) Water and sucrose solution intake of unstressed male rats before and after a 3-week treatment with the combination of MTZ plus VLF (5/7.5 mg/kg) or their vehicles. RM two-way ANOVA followed by Tukey test, ***P < *0.001* sucrose + treatment vs. sucrose + vehicles, *n* = 8 per group

After three weeks of the CMS protocol, all males reduced their sucrose intake; however, this reduction did not meet the anhedonia criterion and were therefore exposed to the CMS for one additional week. At the end of this time, 16 out of the 24 males met the anhedonia criterion and were included in the experiment in two different groups, one receiving the combination of MTZ plus VLF and the other one their vehicles. After the first week of treatment with the combination of MTZ plus VLF (5/7.5 mg/kg) or their vehicles, no difference in sucrose intake between groups was found. However, at the end of the second week of treatment, a significant increase in sucrose intake was observed in the antidepressant-treated group, which persisted until the third week of treatment (RM two-way ANOVA: time: F_7,196_ = 22.36, *p* < 0.0001; treatment: F_3,28_ = 71.28, *p* < 0.0001; interaction: F_21,196_ = 9.41, *p* < 0.0001; *p* = ns in week 1; *p* < 0.001 for weeks 2 and 3 after treatment vs. CMS vehicle-treated controls) (Fig. 2a). No difference in sucrose intake was found in males after the third week of antidepressant treatment as compared to their initial basal intake (Tukey’s, NS). Conversely, the stressed animals treated with vehicle showed very little sucrose intake.

Male rats not exposed to the CMS receiving either vehicles or the combination of MTZ and VLF showed no changes in fluid (sucrose or water) intake (RM two-way ANOVA: time: F_6,84_ = 1.025, NS; treatment: F_1,14_ = 1.96, NS; interaction: F_6,84_ = 1.06, NS) (Fig. 2b) throughout the 6 weeks of evaluation.

### Female rats

**Fig. 3:**
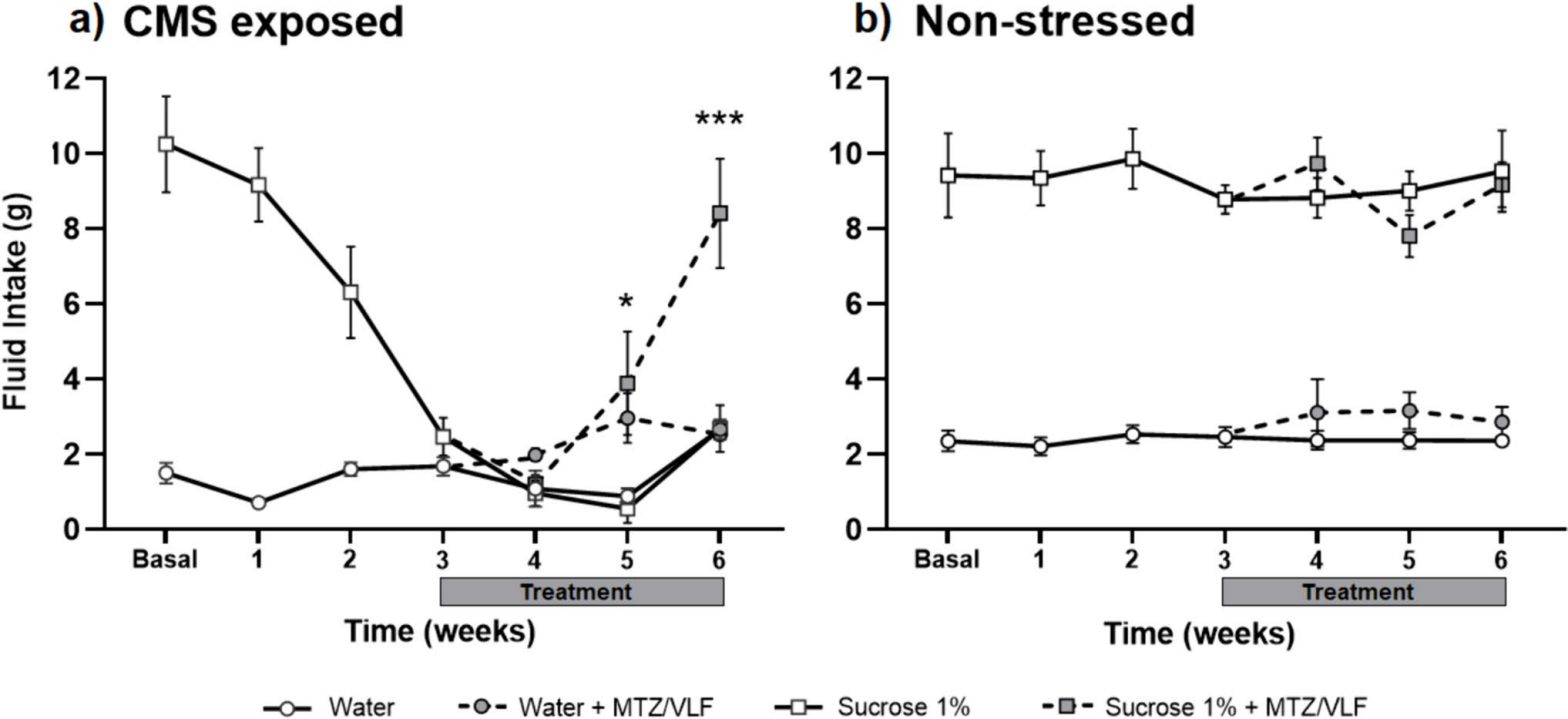
Sucrose intake of female rats that developed anhedonia after CMS in response to MTZ/VLF combined treatment. **a**) Female rats’ water and sucrose solution intake during CMS exposure before and after a 3-week treatment with the combination of MTZ plus VLF (5/7.5 mg/kg) or their vehicles. **b**) Water and sucrose solution intake of unstressed female rats before and after a 3-week treatment with the combination of MTZ plus VLF (5/7.5 mg/kg) or their vehicles. RM two-way ANOVA followed by Tukey test, *P < 0.05; ***P < *0.001* sucrose + treatment vs. sucrose + vehicles, *n* = 8 per group

After three weeks of exposure to the CMS, 16 out of 26 females fulfilled the criterion for anhedonia. The 16 animals that developed anhedonia were divided into two groups, one received the combined treatment with MTZ and VLF (*n* = 8) and the other received the antidepressant vehicles (*n* = 8) for 3 weeks. After one week of treatment no effect on sucrose intake was observed; however, after two weeks a slight increase in the preference for sucrose solution was registered in the group of rats receiving the antidepressant combination (Tukey’s, *p* < 0.05 vs. stressed CMS group treated with vehicles), which was even larger at the end of the third week (*p* < 0.0001), reaching baseline sucrose intake levels (Tukey’s, NS). The statistics showed a significant difference between factors and their interaction (RM two-way ANOVA: time: F_6,84_ = 18.75, *p* < 0.0001; treatments: F_1,14_ = 10.55, *p* = 0.005; interaction: F_6,84_ = 2.88, *p* = 0.01) (Fig. 3a). Female rats subjected to the CMS that were injected with the antidepressant vehicles did not recover the sucrose preference.

In the groups of females that were not exposed to CMS, sucrose and water intake remained stable during the six weeks of the experiment, regardless of the treatment received. This result confirmed that the combination of MTZ and VLF did not modify fluid intake (RM two-way ANOVA: time: F_6,84_ = 0.763, NS; treatments: F_1,14_ = 0.005, NS; interaction: F_6,84_ = 0.599. NS) (Fig. 3b).

The comparison of the antidepressant-like effect of the combination of MZT and VLF between sexes yielded interesting results. No difference between sexes were found in sucrose consumption before and after antidepressant treatment (t-test, NS) in the stressed animals. The value of sucrose intake in the stressed females at the beginning of the treatment (week 3) was 4.11 ± 0.42 g increasing to 8.41 ± 1.45 g at the end of treatment (week 6), while in males, these values were 2.33 ± 0.12 g at the beginning of treatment (week 4) increasing to 6.98 ± 0.50 g at the end of treatment (week 7). By contrast, the time course of the antidepressant-like effect of this combination showed differences between sexes. In males, after two weeks of treatment (week 6) sucrose solution consumption was similar to basal intake (Fig. 2a, Tukey, NS), while in females an increase in sucrose consumption was observed after two weeks of treatment (week 5) which, however, still differed from the basal sucrose intake (Fig. 3a, Tukey *p* < 0.001) and required 3 weeks to be similar to basal levels (Tukey, NS).

*Experiment 3. Analysis of the sexual behavior after chronic treatment with the combination of mirtazapine and venlafaxine in male and female rats*

### Male rat sexual behavior

To assess the putative effects of this antidepressant combination on male rat sexual behavior we used sexually experienced males. All treated animals ejaculated at least twice (range 2—5 ejaculations) during the 30-min test. For this reason, the sexual behavior parameters were determined for the first two copulatory series.

**Fig. 4:**
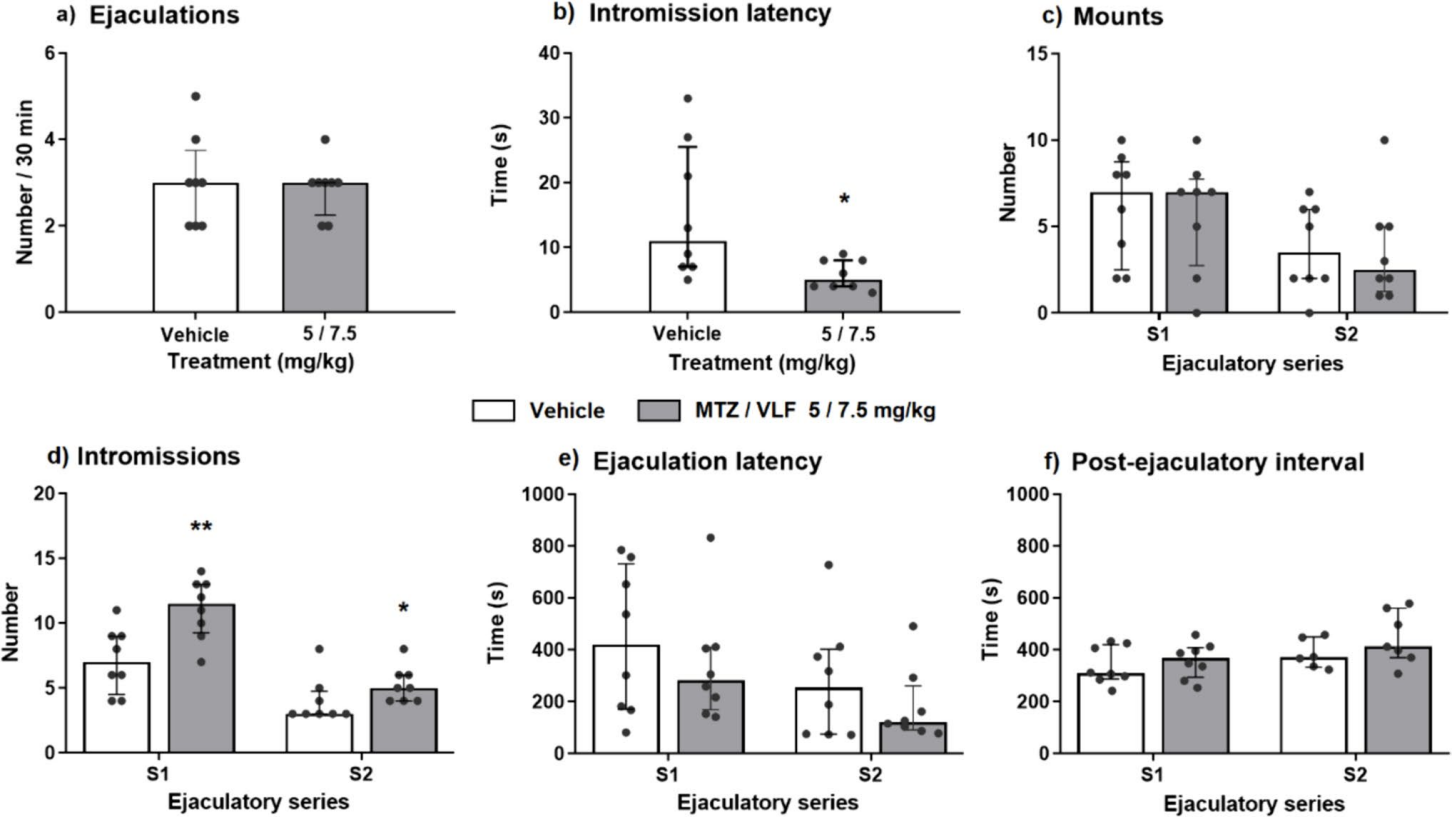
Sexual behavior of male rats chronically treated with a combination of MTZ plus VLF. Specific sexual behavior parameters of sexually experienced male rats treated for 3 weeks (21 days) with MTZ plus VLF (5/7.5 mg/kg) or their vehicles. S1 first series, S2 second series. Data are expressed as medians ± interquartile ranges. **a**) Number of ejaculations in 30 min, **b**) intromission latency, **c**) number of mounts, **d**) number of intromissions, **e**) ejaculation latency, **f**) post-ejaculatory interval. Mann–Whitney *U* test, *P < 0.05; **P < *0.01, n* = 8 per group

When administered for 3 weeks (21 days), the combination of MTZ and VLF did not modify the number of ejaculations achieved in 30 min in comparison with the vehicle-treated group (U = 32, NS) (Fig. 4a). By contrast, the intromission latency was reduced (U = 10.5; *p* = 0.023) (Fig. 4b) and the number of intromissions was increased in both copulatory series of the drug-treated animals. This increase was higher during the first (U = 7.5; *p* = 0.008) than during the second copulatory series (U = 13; *p* = 0.04) (Fig. 4d). No differences were found in the number of mounts in the first (S1) and second (S2) ejaculatory series (S1: U = 28.5, NS; S2: U = 28, NS) (Fig. 4c) and the ejaculation latencies (S1: U = 28, NS; S2: U = 29, NS) (Fig. 4e) as compared to control males treated with their vehicles.

Since the intromission number was increased in the two copulatory series of the drug-treated males, but no changes were found in the corresponding ejaculation latencies, the inter-intromission interval (III) was calculated. No statistically significant difference was found in the III between antidepressant- and vehicle-treated animals (S1: U = 15; NS; S2: U = 17, NS) (data not shown). Also, the postejaculatory intervals were not modified (S1: U = 28, NS; S2: U = 13, NS) (Fig. 4f). It is worth mentioning that the postejaculatory interval of the second ejaculatory series was calculated based on seven animals of the treated group, and six animals of the vehicle group, given that only these rats resumed copulation after the second ejaculation.

### Female rat sexual behavior

**Fig. 5:**
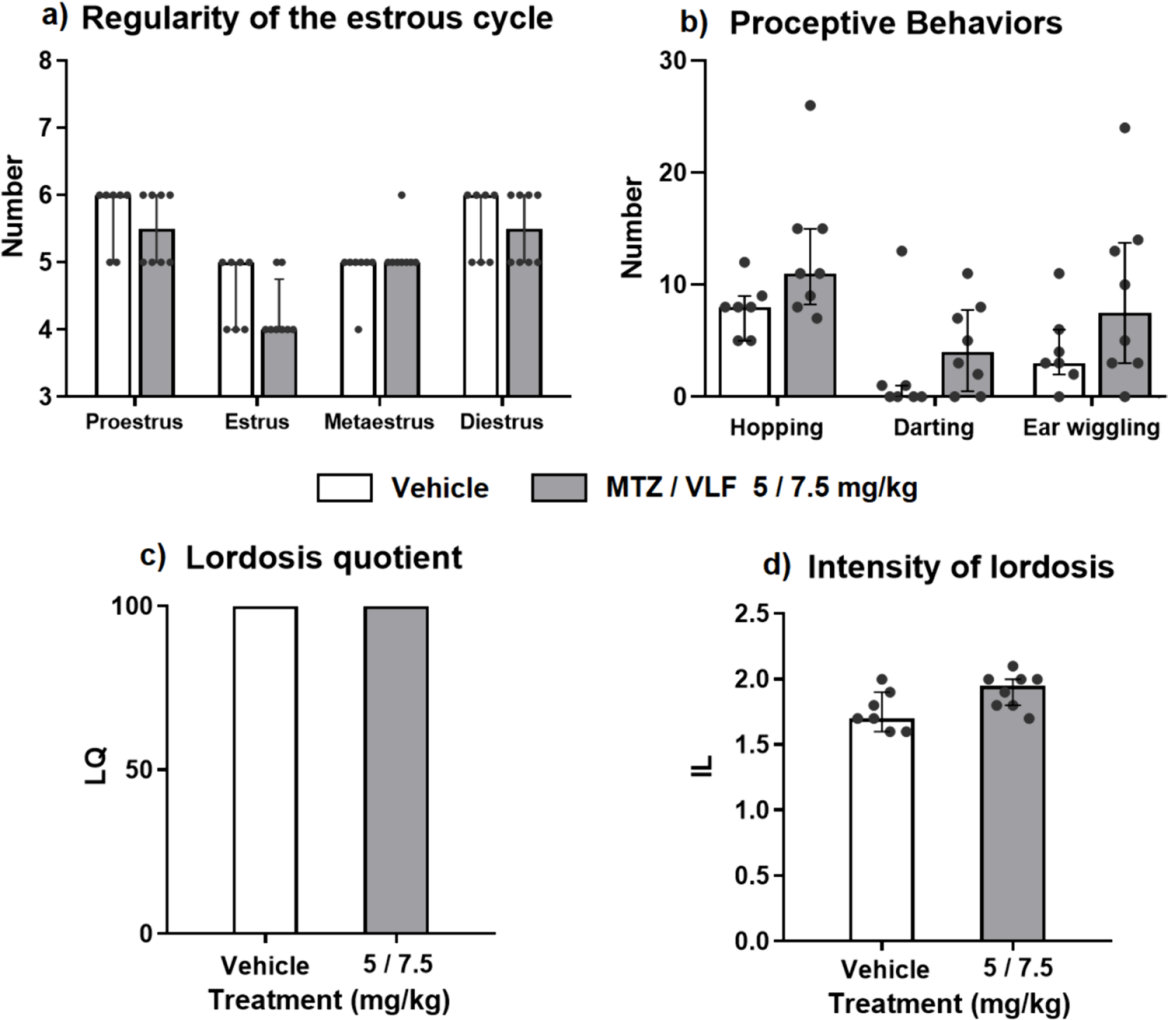
Sexual behavior of female rats chronically treated with a combination of MTZ plus VFL**. a**) Median number ± interquartile ranges of the different estrous phases and **b**) of the distinct proceptive behaviors (hopping, darting and ear-wiggling) exhibited by sexually receptive female rats treated with MTZ plus VLF 5/7.5 mg/kg or their vehicles for 21–24 days. **c**) Lordosis quotient and **d**) lordosis intensity, expressed as median number ± interquartile ranges of MTZ/VLF or vehicle-treated females. Mann–Whitney *U* test NS, *n* = 8 per group

All female rats presented regular 4–5-days estrous cycles along 14 days before the administration of the antidepressant treatment or their vehicles (data not shown). No differences were found in the number of the different estrous cycle phases exhibited by control and drug-treated females (Fig. 5a); that is, the estrous cycle regularity remained unaltered by this antidepressant combination, allowing the evaluation of female sexual behavior under natural conditions, i.e., during the late proestrus phase (Fig. 5a).

Figure 5b compares the median number of proceptive behaviors (hopping, darting, and ear wiggling) between control and antidepressant-treated sexually receptive females during proestrus. No changes in the number of these proceptive behaviors were found after the treatment with the antidepressant combination (hopping: U = 12, NS; darting: U = 16, NS; ear wiggling: U = 17.5, NS).

Receptivity also remained unaltered after the treatment with the combination of MTZ plus VLF. All female rats (control and treated) presented a lordosis quotient of 100 (U = 28, NS) (Fig. 5c). In line, the intensity of lordosis did not differ between vehicle and antidepressant-treated females (U = 12, NS) (Fig. 5d).

## Discussion

The results of this study show that the combined chronic treatment with low doses of mirtazapine and venlafaxine (5 and 7.5 mg/kg, respectively) produced an antidepressant-like effect in both the forced swim test and the chronic mild stress models and lacked an action on male and female rat sexual behavior. Previously, we described that this combination produced an antidepressant-like effect when given sub-chronically (Alvarez Silva and Fernández Guasti 2019), supporting the notion that sub-chronic treatments are as effective as chronic ones to produce antidepressant-like actions (Rénéric et al. 2002b). In this study, we also report that the antidepressant combination was effective in male and in gonadally intact females, like its effect in ovariectomized, steroid-primed female rats (Alvarez Silva and Fernández Guasti 2019). Furthermore, present data showed that lower doses of this antidepressants’ combination (MTZ, 2.5 mg/kg and VLF, 3.75 mg/kg), even when chronically administered, failed to produce an effect in the FST.

### Antidepressant-like effect of mirtazapine plus venlafaxine in male and female rats

#### FST

As previously mentioned, the individual antidepressant-like effects of either MTZ or VLF have been reported earlier, though employing higher doses (Rénéric and Lucki 1998; Rénéric et al. 2002a, 2002b; Rogóz et al. 2002; Alvarez Silva and Fernández Guasti 2019; 2020; Barbar et al. 2022). The antidepressant-like effect of the combination of MTZ plus VLF has, however, not been extensively studied. It is worth mentioning that when combined, much lower doses of each drug were required to produce an antidepressant-like effect. A previous study showed that the lowest effective doses of this combination were one eighth of those required individually to attain the same antidepressant-like effect both in male and female rats (Alvarez Silva and Fernández-Guasti 2019). It is worth mentioning that the MTZ/VLF combination (5/7.5 mg/kg) produced a reduction in spontaneous activity, however, the treated rats exhibited more active behaviors (swimming and climbing) in the FST, reinforcing the specific antidepressant-like effects of this combination (Slattery and Cryan 2012). Currently, when the combination of MTZ and VLF is prescribed in clinical practice, the doses used are the same as those prescribed for each antidepressant as monotherapy. The findings of this study could serve as background to look for a therapeutic effect of lower doses of the combination of MTZ and VLF in humans, potentially reducing negative side-effects.

In the FST the combination of MTZ and VLF produced a similar antidepressant-like effect in male and female rats, but a differential one on active behaviors. In males, the antidepressant combination increased both swimming and climbing behaviors, whereas in females only swimming behavior was enhanced. This sex-difference was also observed after the sub-chronic treatment with this combination and could be related to the estrogens’ effects favoring MTZ and VLF actions at the serotonergic system (see Alvarez Silva and Fernández-Guasti 2020).

### Chronic mild stress

Chronic treatment with 5/7.5 mg/kg MTZ plus VLF reversed the CMS-induced reduction in sucrose consumption in both sexes. The antidepressant combination did not increase fluid intake in the non-stressed groups, evidencing that it lacked unspecific dipsogenic effects. Therefore, the increase in sucrose intake in the stressed groups seems to specifically reflect a reversal of the CMS-induced anhedonia.

Sex differences in the CMS were found. Firstly, male rats did not respond to the current mild stressors used in females, and therefore some stressors as well as their duration had to be modified to induce anhedonia in males. These data agree with previous studies reporting that young adult male rats are less susceptible to stress-induced anhedonia than aged males or young or old females (Herrera-Pérez et al. 2008; Xing et al. 2013; Lu et al. 2015; Vieira et al. 2018). Secondly, the treatment with the combination of MTZ and VLF produced a similar final effect after 3 weeks in males and females. Male rats began to increase sucrose intake after one week of treatment; a similar latency has been reported for a broad range of antidepressants (Willner et al. 1987). In females, after two weeks of treatment there was a slight increase in sucrose intake that reached pre-stress values after three weeks of treatment. That is, the increase in sucrose intake to reach pre-stressed levels took longer in females than in males. In line with these results, previous data showed that the chronic treatment with VLF alone reversed the decrease in sucrose intake produced by CMS with a shorter latency in male than in female rats (Xing et al. 2013). In clinics, there is also evidence that women require a longer treatment with VLF, as compared to men, to show remission of anxiety symptoms (Roca et al. 2008). After these data it could be proposed that the longer latency to recover the basal levels of sucrose intake observed in females, after the combination of MTZ plus VLF, is due to this last antidepressant, given that for MTZ no sex differences in its onset of action have been reported. Interestingly, a significant sex difference, favoring men, was found in the frequency of prescribing MTZ for the treatment of depression, explained by its lack of sexual adverse reactions (Rothschild 2000; Schwalsberger et al. 2022).

The reduction of sucrose consumption in this model is accompanied by a decrease in brain monoamine levels that is reversed by SSRIs (Wang et al. 2017). The data of this investigation do not allow to determine the mechanism by which the MTZ/VLF combination produced the antidepressant-like effect; however, it could be speculated that given that this combination exerts a synergistic action at serotonergic, noradrenergic and dopaminergic systems (de la Gándara et al. 2005), a similar mechanism might underly it. Moreover, chronic treatment with either MTZ or VLF in rats reverses the effects of CMS on anhedonia, on GABA reduction, and on glutamate and BDNF increases (Zhang et al. 2010; Kamal 2013). Similar changes in brain GABA, glutamate and BDNF have been found in patients with depression, and their reversal appears to play an important role in the improvement of depressed mood (Sanacora et al. 2003; Lee and Kim 2010; Molendijk et al. 2014). Interestingly, chronic VLF has also been reported to reduce S100B protein expression, a marker of glial activity that has been found to be increased in depressed patients (Wang et al. 2016).

## Effect of mirtazapine plus venlafaxine on sexual behavior

### Males

To our knowledge this is the first study reporting on the effects of MTZ and VLF combined on male rat sexual behavior. Present results showed that the chronic administration of the combination of MTZ plus VLF reduced the intromission latency and increased the number of intromissions necessary to achieve ejaculation in two successive copulatory series but had no effects on the other parameters. The reduction in the intromission latency suggests a facilitatory effect of this combination on sexual motivation (Beach 1956). On the contrary, the increased number of intromissions may be interpreted as a male’s higher requirement of penile stimulation to achieve ejaculation. Thus, antidepressant-treated males did not show sexual behavior impairments.

To remind, MTZ or VLF, administered separately, alter male rat sexual behavior. Benelli et al. (2004) reported an enhanced sexual motivation, after the chronic administration of MTZ, while VLF negatively affected sexual behavior by reducing the ejaculation frequency (Bijlsma et al. 2014). These data suggest opposite effects of MTZ and VLF on male rat sexual behavior and permit to propose that the absence of significant effects on sexual activity after their combined treatment might result from a balance between the sexual facilitatory (MTZ) and the sexual inhibitory (VLF) actions. In support of this proposal, the sexual inhibitory effects of fluoxetine are attenuated by added-on mirtazapine (Benelli et al 2004). A similar effect has been reported in men, where the addition of MTZ reverses SSRI-induced sexual dysfunction (Farah 1999; Ozmenler et al. 2008; Atmaca et al. 2011). This reversal might be associated to the 5-HT2 receptor antagonist action of MTZ, since this receptor is related to the development of SSRI-induced sexual dysfunction (Klint and Helsdingen 1996; Alcántara 1999; Zemishlany and Weizman 2008). Another possibility is that the low dose of VLF here used is subthreshold to impair male sexual behavior.

### Females

The combination of MTZ and VLF used in the present study did not modify the regularity of the estrous cycle, permitting the sexual behavior evaluation in naturally cycling female rats (during late proestrus). This combined treatment also lacked an effect on female sexual behavior.

There are several studies on the effect of antidepressants on the regularity of the estrous cycle. Thus, chronic treatment with high doses of fluoxetine was reported to disrupt the estrous cycle by increasing the occurrence of the metestrus phase (Domingues et al. 2023), while lower fluoxetine doses lacked an action (Van de Kar et al. 2002) or produced only a modest effect (Maswood et al. 2008). In women, no effects of antidepressant treatment, independently of their class, on the regularity of the menstrual cycle have been reported (Casilla-Lennon et al. 2016).

Both MTZ and VLF, given independently at doses with antidepressant-like effects, inhibit female rat sexual behavior. Thus, VLF reduced proceptive behaviors without affecting lordosis, while MTZ, markedly diminished both proceptivity and receptivity (Alvarez Silva and Fernández-Guasti 2019). However, low doses of MTZ and VLF combined did not affect proceptivity or receptivity in ovariectomized, steroid-primed female rats (Alvarez Silva and Fernández-Guasti 2019) or intact (present results) animals.

A possible explanation for the lack of sexual effects of this combination is that the MTZ-mediated serotonin increases, that accounts for the inhibition of female sexual behavior (Uphouse et al. 2009; Uphouse 2014), is counteracted by the addition of VLF that increases dopamine and noradrenaline (Millan et al. 2001). Another possibility is that the low doses of MTZ and VLF are subthreshold to modify female sexual behavior, even if they were effective in reducing depressive-like behaviors (Alvarez Silva and Fernández-Guasti 2019).

As aforementioned the most relevant issues for patients receiving antidepressant treatment are their failure to reduce depressive symptoms and their negative effects on sexual function. These caveats difficult the adherence to treatment, affecting long-term prognosis. The results of this study suggest that the combination of low doses of MTZ and VLF might be a promising therapeutic alternative to treat depression without affecting the sexual response.
